# Pitfalls in Neuroimaging of Headache: A Case Report and Review of the Literature

**DOI:** 10.1155/2013/735147

**Published:** 2013-02-28

**Authors:** V. Vallamkondu, M. Shakeel, A. Hussain, D. McAteer

**Affiliations:** ^1^Department of Otolaryngology-Head and Neck Surgery, Aberdeen Royal Infirmary, Aberdeen AB252ZN, UK; ^2^Department of Radiology, Aberdeen Royal Infirmary, Aberdeen, UK

## Abstract

Headache is a common symptom, with a lifetime prevalence of over 90% of the general population in the United Kingdom (UK). It accounts for 4.4% of consultations in primary care and 30% of neurology outpatient consultations. Neuroimaging is indicated in patients with red flag features for secondary headaches. The guidelines recommend CT or MRI scan to identify any intracranial pathology. We present a unique case where the initial noncontrast CT scan failed to identify a potential treatable cause for headache. A middle aged man presented with headache and underwent a CT scan without contrast enhancement. The scan was reported as normal. The headache persisted for years and the patient underwent a staging CT scan to investigate an oropharyngeal cancer. This repeat CT scan utilized contrast enhancement and revealed a meningioma. Along with other symptoms, headache is an established presenting complaint in patients with meningioma. The contrast enhanced CT brain proved superior to a nonenhanced CT scan in identifying the meningioma. In a patient with persistent headache where other causes are excluded and a scan is to be requested, perhaps contrast enhanced CT is a better option than a plain CT scan of brain.

## 1. Introduction

Headache is a common symptom, with a lifetime prevalence of over 90% of the general population in the clinical practice [1]. Overall 1-year prevalence of headache in European adults is 51% [2]. It accounts for 4.4% of consultations in primary care and 30% of neurology outpatient consultations. Neuroimaging is indicated in select patients with headache. The guidelines suggest that either a CT or MRI scan can be done to identify any intracranial pathology. We report a unique case where the initial CT scan failed to identify a potentially treatable cause for headache. 

## 2. Case Report

A 74-year-old Caucasian male originally presented to the headache clinic with 1-year history of right sided headache affecting the frontotemporooccipital region. It was daily persistent headache with a pain intensity of 4/10. The nature of pain varied between dull ache with short lived shooting in the right retroorbital area once or twice a week with occasional photophobia. There was no nausea or visual dysfunction. Postural changes or valsalva did not aggravate the headache. The headache failed to settle with analgesics. There was a previous history of migraine in his teens which varied in frequency and severity. The last episode of migraine was experienced 10 years ago. There was a positive family history of migraine with both mother and sister undergoing treatment presently. The patient had significant medical history of ischemic heart disease, diabetes mellitus, and COPD.

There was no clinical abnormality on routine head and neck examination. Detailed neurological examination failed to reveal any abnormality. 

The differential diagnosis included hemicrania continua or chronic migraine. A trial of indomethacin with a starting dose of 25 mg was given and the dose was titrated slowly upward weekly depending on his tolerability and his INR. A non-contrast CT scan of the brain was carried out to rule out intracranial pathology. 

**Figure 1:**
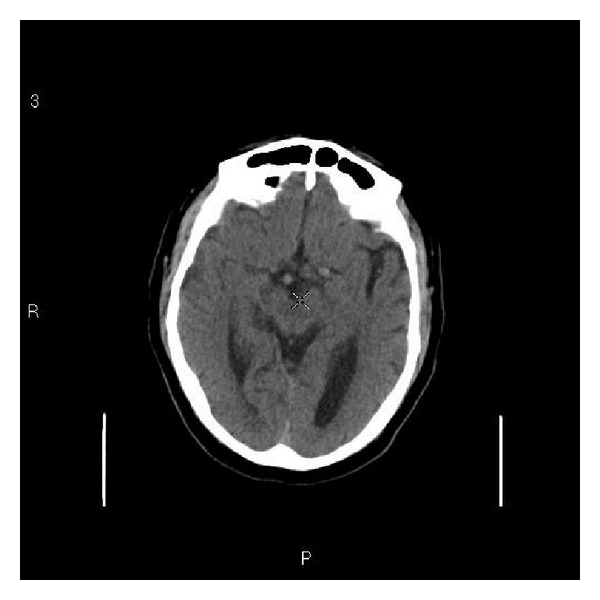
Noncontrast CT scan showing age related atrophy changes.

During the follow-up visits the headache had migrated to the rest of cranium. The CT scan showed age related cerebral atrophy with no intracranial abnormality (Figure 1). Patient was reassured that no serious intracranial abnormality was identified and was treated with a diagnosis of migraine. The response to the standard prophylactic and therapeutic treatment for migraine was suboptimal and variable. 

**Figure 2:**
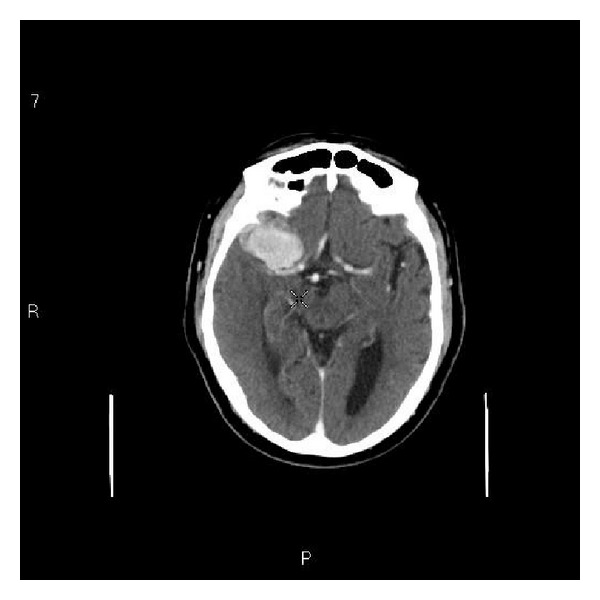
Contrast CT scan showing a 4 cm enhancing mass in the right middle cranial fossa in keeping with a sphenoidal ridge meningioma.

Six months later, the patient presented with new symptoms of right sided odynophagia and dysphagia which were confirmed to be due to the advanced oropharyngeal carcinoma with cervical metastasis. A staging contrast enhanced CT scan of head, neck, and chest was performed which also identified a 4 cm enhancing mass in the right middle cranial fossa in keeping with a sphenoidal ridge meningioma (Figure 2). There was mild surrounding edema but no midline shift was noted. The case was discussed in the multidisciplinary meeting and it was concluded that brain metastasis from the oropharyngeal cancer was unlikely. The patient underwent surgery with postoperative radiotherapy for his oropharyngeal cancer. Because of the serious comorbidity, surgical resection of meningioma was considered too risky and inadvisable. The patient will remain under serial radiological (MRI scan) surveillance for his meningioma. The headache is thought to be secondary to meningioma and pain is managed with standard analgesics.

## 3. Discussion

Headache is one of the most common presenting features in general practice, neurology, or otolaryngology clinics. Headache constitutes a major public health problem and socioeconomic burden.

There are many classification systems for headaches. World Health Organization (WHO) classification was published in Cephalalagia 2004 based on the International Headache Society's revised International Classification of Headache Society's Disorders (ICHD 2) [3]. The British Association for the Study of Headache simplified the ICHD 2 into primary and secondary headaches. Primary headaches are not associated with underlying pathology. Migraine, cluster headache, and tension headaches are grouped as primary headaches. Secondary headache is due to underlying pathology such as infections, vascular causes, trauma, and neoplasms [4].

The aetiology of most headaches can be elucidated by careful history and examination. The diagnosis is further supplemented by judicious use of radiological investigation. If neurological symptoms are present then a brain CT or MRI should be done as a primary investigation [5]. Perhaps contrast enhanced CT is a better option than a plain CT scan of brain. The disadvantages of MRI as a first line investigation are cost, availability, and perhaps most importantly, a very high sensitivity leading to a high detection of indeterminate findings.

In our case, the non-contrast CT failed to diagnose meningioma which is thought to be the underlying cause for his headache. Headache may be noticed in 60% of patients with intracranial tumour but headache may not be the presenting feature. Schankin et al. [6] published data on 58 cases of meningioma, 40% of meningioma patients had associated headache features. Of these, the pain was migraine-like in 22% and tension-type-headache- (TTH-) like in 57%. There is a consensus that mass lesion produces headache either by inducing pressure on pain sensitive dura or arachnoid membranes or by increase in ICP but robust evidence is lacking. Moreover the literature suggests that patients with meningioma present with isolated headache without clinical signs of raised intracranial pressure [7].

In our case there were no neurological symptoms to raise the suspicion of intracranial neoplasm. Therefore he was not given any contrast during neuroimaging. The exact cause of his headache remained unknown. The meningioma was diagnosed on a repeat CT scan with contrast enhancement. Meningioma is a potentially treatable cause of headache. 

## 4. Conclusion

The neuroimaging guidelines for investigation of headache do not recommend contrast enhancement as routine. However, this case highlights the inadequacy of the non-contrast CT scan to diagnose meningioma. In a patient with persistent headache where other causes are excluded and a CT scan is to be requested, the clinician should be aware of the limitations in the sensitivity of an un-enhanced head CT, and where concerning symptoms persist, an alternative imaging modality such as MRI scan should be considered.
